# 

**Published:** 1874-02

**Authors:** 


☞ Don't forget the one thing needful. ☜

Those who desire to compete for the premiums may secure subscribers by written appeals to their friends. They can say to them that, should they send in their names, to state to whom we are indebted for their subscriptions, and each name thus procured shall be counted in the contest for premiums. The money can be sent within sixty days after the reception of the first number. All subscriptions will be commenced with the January number, so long as they last. Send early, or they will be exhausted.

☞ Write your names, Post Office, County and State *plainly*. Be sure to say what Post Office you wish the RECORD sent.

☞ All communications, including remittances of money, etc., must be addressed to POWELL & GOLDSMITH.

Send money by check or Postal Order. Where these cannot be obtained, send at our risk, by registered letters.

As the editors work for "love and not money," spending every dollar made by the Journal on it—they hold no reserve fund with which to pay for articles. All friends of science and humanity are cordially invited to contribute articles for publication. The best we can do for our friends, is as follows:

All subscribers by *remitting* $3.30, will be entitled to Wood's Household Magazine, with a magnificent Chromo of the "Yosemite Valley"—worth, alone, several dollars.

☞ We can furnish a few more bound copies of the RECORD for 1873, at cost, $4.00 a volume.

All subscribers who will send two new subscriber (*besides his own name,*) shall receive one copy of *Powell's Pocket Formulary* or one copy of Wood's Household Magazine, with the superb Chromo of the "Yosemite Valley." Say plainly when remitting the money, which premium you desire.

All subscribers who will forward FIVE CASH SUBSCRIBERS (*besides his own name,*) will be entitled to one copy of the RECORD gratis: or, in lieu thereof, the Formulary and Wood's Household Magazine with Cromo. State clearly what you desire when the five subscribers are procured.

As PRMIUMS, we will furnish *on the first of April next,* a splendid PORTABLE ELECTRO MNGNETIC MACHINE, Manufactured by the "Galvano Faradic Manufacturing Company," of New York, and worth $25.00, to any one sending us *the targest number of cash subscribers* over ten.

The one sending the next largest number over ten, will be entitled to the works of Sir. J. Y. Simpson, consisting of three volumes: Obstetrics and Gynœcology, Diseases of Women, Anæsthesia and Hospitalism.

The one sending the next largest number over ten, will receive "CODMAN & SHURTLIFF'S COMPLETE STEAM ATOMIZER FOR INHALATION.

The one sending the next largest number over *ten,* will be entitled to one copy of the RECORD, POWEL'S FORMULARY AND WOOD'S HOUSEHOLD MAGAZINE, with the large and magnificent CHROMO.

These instruments, books, etc., have been bought and await shipment to the successful persons, on the *first day of April next.* Go to work, friends, and make an effort to secure one of these splendid and useful premiums. This is the best we can do, and we pledge ourselves to comply, in every respect, to the terms above. ALL who do not procure the RECORD upon the several terms above given, are expected to PAY THE FULL SUBSCRIPTION PRICE.

*Dr. B. E. Reese's letter*, with editorial comment, will appear in our next.

D. Appleton & Co., of New York, have sent us a new work, entitled, An Introduction to Physical Measurements, etc., by Dr. F. Kohlrausch, which will be noticed in our next.

*Correspondents and others* sending communications will confer a favor upon the printer by writing very carefully and plainly, *particularly medical technicalities*, and with *ink* rather than pencil.

The following publications, from the well-kown publisher, Henry C. Lea, of Philadelphia, came to hand too late for notice in the present number, but will be reviewed in our next. "A clinical history of the Surgical Diseases of Women, by Robert Barnes, M.D., London; and a System of Midwifery, including the Diseases of Pregnancy and the Puerperal State, by William Leishman, M.D."

### LIST OF CASH PAYMENTS TO THE RECORD:

For 1873.

| | |
|---|---|
| Dr. A. Donald | $2 50 |
| Dr. W. A. Harrison, S. C | 2 50 |
| Dr. C. N. Candler, N. C | 2 50 |
| Dr. W. F. Berry, N. C | 2 50 |
| Dr. W. H. Summers, Tennessee | 2 50 |
| Dr. S. G. Hart, Georgia | 2 50 |
| Dr. G. M, Rivers, South Carolina | 2 50 |
| Dr. W. R. Chambers, Alabama | 2 50 |
| Dr. Edward Lea, Mississippi | 2 50 |
| Dr. William Owen, Georgia | 2 50 |
| Dr. O. C. Heery, Georgia | 2 50 |
| Dr. Charles Pinckney, Georgia | 2 50 |
| Dr. Robert Crawford, Georgia | 2 50 |
| Dr. W. C. Asher, Georgia | 2 50 |
| Dr. Mount Scott, Louisiana | 2 00 |
| Dr. C. K. McCord, Alabama | 2 50 |

For 1874.

| | |
|---|---|
| Dr. J. W. Bennett, North Carolina | 2 50 |
| Dr. W. J. McLendon, North Carolina | 2 50 |
| Dr. O. Gregory, North Carolina | 2 50 |
| Dr. J. M. Williamson, Mississippi | 2 00 |
| Drs. Edwards & Allen, Tennessee | 2 50 |
| Dr. W. A. Harrison, South Carolina | 2 50 |
| Dr. A. J. Ellis, North Carolina | 2 50 |
| Dr. J B. Mathews, Georgia | 2 50 |
| Dr. Alex. Brown, Georgia | 2 50 |
| Dr. J. Gus Sims, Tennessee | 2 50 |
| Dr. J. W. Allen, Tennessee | 2 50 |
| Dr. J. C. Knox, Alabama | 2 50 |
| Dr. T. C. Powel, North Carolina | 2 50 |
| Dr. R. A. Sills, North Carolina | 2 50 |
| Dr. J. H. Knott, Mississippi | 2 50 |
| Dr. W. S. Posey, Texas | 2 50 |
| Dr. H. B. Griffith, Mississippi | 2 50 |

For 1874.

| | |
|---|---|
| Dr. J. W. Ethridge, Mississippi | $2 50 |
| Dr. N. B. Warren, Mississippi | 2 50 |
| Dr. John Mars, Mississippi | 2 50 |
| Dr J. D. Kirk, Mississippi | 2 50 |
| Dr. James Rogers, Mississippi | 2 50 |
| Dr. J. A. Hanks, North Carolina | 2 50 |
| Dr. G. S. P. Brown, North Carolina | 2 50 |
| Dr. John Gerdine, Mississippi | 2 50 |
| Dr. Archibald Palmer, North Carolina | 2 50 |
| Dr. B. M. Walker, North Carolina | 2 50 |
| Dr. N. B. Richards, Illinois | 2 50 |
| Dr. W. H. Summers, Tennessee | 2 50 |
| Dr. S. G. Hart, Georgia | 2 50 |
| Dr. T. P. Oliver, Georgia | 2 50 |
| Dr. P. W. Calhoun, Louisiana | 3 00 |
| Dr. R. S Hallsey, North Carolina | 2 50 |
| Dr. Hale, New Jersey | 1 00 |
| Dr. Jos. Underwood, Georgia | 2 50 |
| Dr. H. L. Petrie, Mississippi | 2 50 |
| Drs. Slansill & Robbins, N. C | 2 50 |
| Dr. C. B. Nottingham, Georgia | 2 50 |
| Dr. P H Wright, Georgia | 2 50 |
| Dr. J. S. Davis, Louisiana | 2 50 |
| Dr. J. S. Green, Mississippi | 2 50 |
| Dr. J. R. Green, Mississippi | 2 50 |
| Dr. J. W. Gilbert, Alabama | 2 50 |
| Dr. T. L. Quillian, Alabama | 2 50 |
| Dr. L. S. Brownlee, Mississippi | 2 50 |
| Dr. J. E. Carter, Georgia | 2 50 |
| Dr. F. M. Rushing, Alabama | 2 50 |
| Dr. W. Barton, Georgia | 2 50 |
| Dr. Robert T. Barton, Georgia | 2 50 |
| Dr. Lockwood Alison, Louisiana | 2 50 |
| Dr. Hartwell Alison, Louisiana | 5 50 |
| Dr. B. J. A. Cull, Georgia | 2 50 |
| Dr. W. J. Hayes, Mississippi | 2 50 |

The above list of names are those who have paid for volume 73 and 74. If we have failed to credit any, they will do us the favor to inform us of the fact. *February* 23, 1874.

### ASSOCIATE EDITORS:

GEORGIA.

James A. Long, M.D.

W. L. M. Harris, M.D.

H. L. Battle, M.D.

W. C. Musgrove, M D

L B. Bouchelle, M.D.

J. Q. A. Hutson, M.D.

C. H. Tidd, M.D.

### CORRESPONDING EDITORS:

Ely Van DeWarker, M. D., New York.

C. C. F. Gay, M.D., New York,

*Surgeon of Buffalo General Hospital.*

M. H. Henry, M.D., New York,

*Surgeon to N. Y. Dispensary, Dep't of Venerial and Skin Diseases.*

Benjamin Howard, M.D., New York.

James Tyson, M.D., Philadelphia, Pa.

W. W. Keen, M.D. Philadelphia.

Geo. Strawbridge, M.D., Philadelphia.

Robert Robson, M D., Indiana.

A. Patton, M.D., Indiana.

John H. Weir, M.D., Illinois.

Thomas J Gallaher, M. D, Pennsylvania.

S. C. Busey, M.D., Washington, D. C.

Wm. R. Whitehead, M.D., Colorado Territory.

